# A Simple Treatment of Bronchial Asthma

**Published:** 1914-01

**Authors:** 


A Simple Treatment of Bronchial Asthma. Gruenwald *Muench. med. Wochenschr.,* No. 25, 1913. By means of a laryngeal syringe, 1 c. cm. of a 1:10,000 solution of suprarenin (adrenalin) is injected through the glottis into the trachea. The patient immediately feels a sensation of cold, extending down into the epigastrium, and, soon after, the respiration becomes less difficult. A single injection apparently suffices to abort the attack, and indeed, in the writer's experience, to prevent its recurrence for several months. The treatment is most successful in the so-called idiopathic bronchial asthma; it fails in reflex asthmatic attacks and in the asthma of hay-fever.

